# Eosinophilic granulocytes as a potential prognostic marker for cancer progression and therapeutic response in malignant melanoma

**DOI:** 10.3389/fonc.2024.1366081

**Published:** 2024-05-02

**Authors:** Corsin Linard Brand, Robert Emil Hunger, Seyed Morteza Seyed Jafari

**Affiliations:** Department of Dermatology and Venerology, University Hospital of Bern, University Bern, Bern, Switzerland

**Keywords:** cancer, eosinophils, immune checkpoint inhibitors, immunology, melanoma

## Abstract

The importance of eosinophilic granulocytes in cancer has been widely discussed in recent years. The current study reviews the evidence on the role of eosinophilic granulocytes in melanoma as a prognostic marker for cancer progression and the efficacy of treatment with modern immune checkpoint inhibitors. A total of 33 human clinical studies were included in the review, with heterogeneous data due to differences in patients populations, study design and inclusion of small study groups. However, 28 of the 33 studies suggested that eosinophilic granulocytes could be used as a prognostic biomarker for outcome and/or potential response to systemic treatment and/or occurrence of adverse events in melanoma patients. Nevertheless, the exact role of eosinophils remains to be elucidated. Further prospective, larger and better controlled studies are warranted to clarify the significance of eosinophilic granulocytes in patients with melanoma, in more details.

## 1 Introduction

In contrast to many other cancers, the global prevalence of melanoma continues to increase (1, 2). Early detection is crucial for a successful treatment (1). In recent years, there have been significant developments in therapeutic options. Immune checkpoint inhibitor (ICI) therapies, such as anti-cytotoxic T-lymphocyte-associated protein 4 (CTLA4) and anti-programmed cell death protein 1 (PD1), have shown promising results (3).

In the era of personalized medicine, there is great interest in finding prognostic markers that can predict survival, outcome, or response to therapy (4). The potential prognostic biomarkers in melanoma regarding overall survival (OS) are melanoma-inhibitory activity (MIA), S100 protein, lactate dehydrogenase (LDH), and possibly eosinophilic granulocytes (5–7). In the last decade, the role of eosinophils in malignant melanoma has been increasingly discussed. Eosinophilic granulocytes, identified histologically by their acidophilic staining pattern and heavy cytoplasmic granules, are primarily recognized for their immune function against helminths, parasites and during an allergic reactions (8–13). In addition, eosinophils may play a role in modulating the tumor microenvironment (TME) and immune response, and probably influencing the outcome of ICI therapies in melanoma patients, making them a potential biomarker to predict response to therapy. In the present study, we focused on the current knowledge of the role of eosinophilic granulocytes as a potential prognostic marker for melanoma progression, with a focus on the efficacy of treatment with modern ICIs.

## 2 Methods

This review was conducted based on the PRISMA group statement. The systematic literature search was performed in the PubMed Library from January 2000 to December 2023 using the following search terms or respective combinations: “melanoma [Title] AND (eosinophils or eosinophil)” and “melanoma [Title] AND (eosinophils or eosinophilic or tumor associated blood eosinophilia (TABE) or tumor associated tissue eosinophilia (TATE) or tissue eosinophilia (TE)) AND (prognosis or prognostic or outcome or overall survival (OS))”. The publication had to be a human study. Review articles, case reports, case series (with fewer than 15 patients), meta-analyses, and animal studies were excluded.

## 3 Results

**Figure 1:**
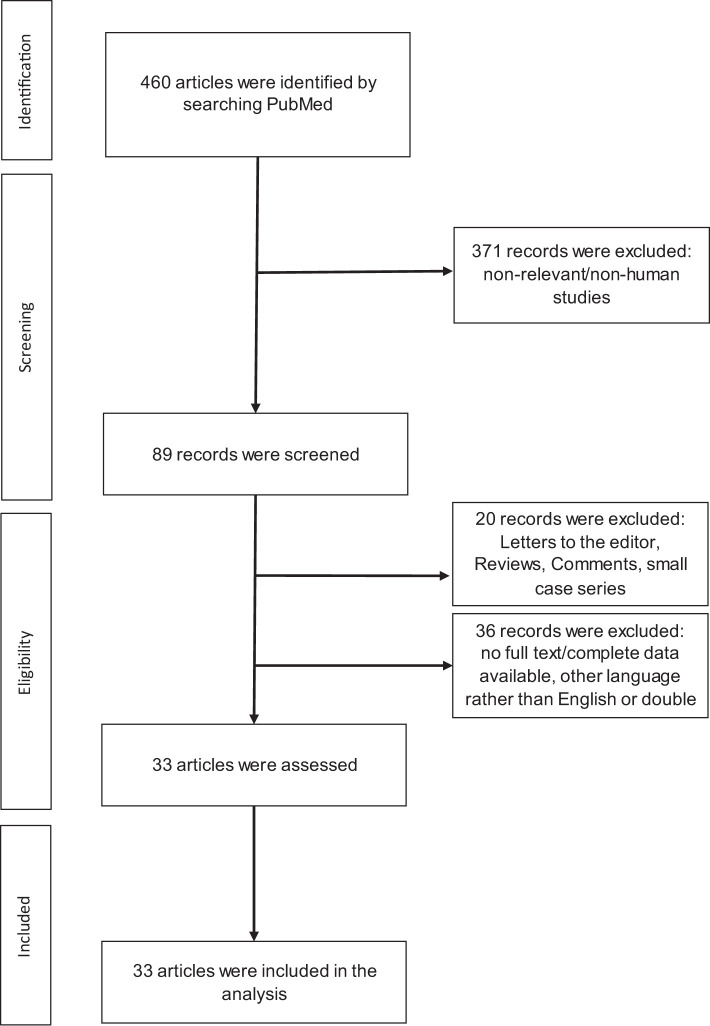
Flow of information through the different phases of the review.

A total of 460 articles were initially identified. After a thorough review and screening of all abstracts, 427 articles were excluded. Finally, 33 clinical studies met the search terms and inclusion criteria, as shown in **Figure 1**. A summary of the selected publications is presented in **Table-1**, in chronological order. Most of the studies were retrospective in design. 28 of the 33 studies suggested that eosinophilic granulocytes could be used as a prognostic biomarker for outcome and/or potential response to systemic treatment and/or occurrence of adverse events in melanoma patients.

**Table 1:** Overview of all the included publications.

No.	Study/Study Type	Patients	Results
1	Tasaki et al., 2023 (14) Retrospective study	614 patients with cancer (melanoma, n=64)	Elevated eosinophils prior to two courses of treatment may be a predictor of immune-related adverse events in various cancers treated with different immune checkpoint inhibitors.
2	Pozorski et al., 2023 (15) Retrospective study	183 patients with unresectable stage III- IV melanoma treated with anti-PD-1 monotherapy (nivolumab or pembrolizumab) or combination ipilimumab/nivolumab	The baseline neutrophil/eosinophil ratio may be a novel prognostic marker for advanced melanoma patients receiving anti-PD-1-based therapies.
3	Goldschmidt et al., 2023 (16) Retrospective study	18186 metastatic solid tumors (melanoma, n = 3314)	Better OS correlated with increased baseline serum albumin concentration, increased eosinophil and lymphocyte counts.
4	Mehra et al., 2023 (17) Retrospective study	229 patients with different tumour entities (melanoma, n=66)	The study found an independent association between the occurrence of immune-related adverse events and improved overall survival in a real-world cohort across multiple tumor types and treatment regimens. Pre-treatment comorbidities, CRP and eosinophil count are potential markers for predicting treatment response.
5	Obashi et al., 2022 (18) Retrospective study	16 patients with unresectable malignant melanoma (stage III-IV), received nivolumab (n=12) or pembrolizumab (n=4)	No significant difference was found in the baseline value of relative neutrophil count, relative lymphocyte count, neutrophil to lymphocyte ratio, and relative eosinophil count between responders and non-responders. However, responders after anti-PD-1 therapy revealed the increase of lymphocytes and eosinophils and the decrease of neutrophils within the first 6 weeks of the treatment.
6	Rafei-Shamsabadi et al., 2022 (19) Retrospective study	27 patients with stage III-IV (59%) melanoma who showed locoregional progression under previous immunotherapy with PD-1-inhibitors, received additive intrelesional interleukin-2	Prolonged PFS and OS were significantly associated with an increase in absolute peripheral blood eosinophil count during IL-2 treatment.
7	Ammann et al., 2022 (20) Retrospective study	285 primary or metastatic tumor tissue specimens from 118 cutaneous melanoma patients of all stages, received pembrolizumab, nivolumab, or a combination of nivolumab and ipilimumab	Positive correlation between increased tumor-infiltrating eosinophils and T-cells associated with delayed melanoma progression was observed. High baseline levels of eosinophil count, serum eosinophil cationic protein and eosinophil peroxidase were linked to prolonged progression-free survival in metastatic melanoma receiving immune checkpoint inhibition.
8	Kurzhals et al., 2022 (7) Retrospective study	46 patients with stage III-IV melanoma, received adjuvant immunotherapy with either nivolumab or pembrolizumab	Baseline lymphocyte and eosinophil counts and those during immunotherapy were not associated with disease recurrence.
9	Wendlinger et al., 2022 (21) Prospective and retrospective study	94 patients with advanced malignant melanoma received dual targeted therapy. 112 patients, received immunotherapy served as control cohort	High pre-treatment eosinophil counts in advanced melanoma patients were associated with a significantly improved response to MAPK signaling pathway inhibitors (MAPKi). Functionally, eosinophils show potent cytotoxicity towards melanoma cells, which can be reinforced by MAPKi.
10	Diab et al., 2021 (22) Phase II cohort	41 previously untreated patients with stage III/IV melanoma received bempegaldesleukin Plus nivolumab	Early on-treatment blood biomarkers (CD8^+^ polyfunctional strength difference and eosinophils) correlated with treatment response. An early on-treatment increase in eosinophils correlated with a higher objective response rate but not with PFS.
11	Kartolo et al., 2021 (23) Retrospective study	86 patients with advanced melanoma on PD-1 inhibitors	Eosinophilia-on-immunotherapy and its timing were associated with better immunotherapy efficacy in patients with advanced melanoma. Our findings provided insights on potential therapeutic benefit of inducing eosinophilia at certain interval time to obtain a longer durable immunotherapy response.
12	Machiraju et al., 2021 (24) Retrospective study	113 patients with advanced melanoma who received treatment with anti-PD1 (47 pembrolizumab 1 nivolumab), anti-CTLA4 (23 ipilimumab) or anti-CTLA4 plus Anti-PD1 (42 ipilimumab plus nivolumab)	There was a significant increase in the absolute eosinophils in blood under combination treatment and anti-CTLA-4 treatment but not upon anti-PD1 monotherapy.
13	Zhang et al., 2021 (25) Analysis of TCGA* database	80 patients with uveal melanoma in the TCGA database	The patients with choroidal melanoma were divided in two immune subgroups of tumor microenvironment. Class1 has low immune infiltration, contains memory B-cells, T helper-2 cells, T helper-17 cells, natural killer cells and eosinophilic granulocytes, and has a better prognosis. CD8^+^ T cells, T helper-1 cells, myeloid-derived suppressor cells, and dendritic cells are enriched in class2, which has strong cytolytic activity, high expression of immune checkpoint genes, and poor outcome.
14	Bai et al., 2021 (26) Analysis of the patients from two prospective trials	89 patients with advanced Melanoma (stage IV), received anti-PD1 monotherapy	Low early-on-/pre-treatment fold change of eosinophil was associated with a poor PFS. Pre-treatment eosinophil count was significantly negatively associated with OS. Low early-on-/pre-treatment fold change of eosinophil was not significantly associated with a better response to treatment.
15	Simon et al., 2020 (27) Prospective study	32 patients with unresectable stage III or IV melanoma, received pembrolizumab (n=22) or the combination of nivolumab/ipilimumab (n=10)	Clinical responses to ICIs treatment were associated with an eosinophil accumulation in the peripheral blood. This finding highlights additional mechanisms of ICIs effects and suggest the level of eosinophils as a novel predictive marker for melanoma patients who may benefit from the immunotherapy.
16	Wagner et al., 2020 (28) Retrospective study	1412 patients with stage I-II melanoma	Absolute eosinophils ≤200/µL and relative eosinophils ≤2.7% were significantly associated with reduced OS in one cohort each. A combined score including absolute levels of neutrophils lymphocytes, monocytes and eosinophils was significantly associated with OS in both cohorts. Analysis of distant metastasis-free survival revealed similar results.
17	Balatoni et al., 2020 (29) Retrospective study	47 patients with stage III-IV melanoma, treated with ipilimumab	Baseline absolute eosinophil counts > 0.1 G/L was significantly associated with diminished progress free survival. In the routine clinical practice considering performance status, number of affected organs, erythrocyte sedimentation rate, eosinophil count, neutrophil to lymphocyte and eosinophil to lymphocyte ratio beside LDH to identify patients most likely to benefit from ipilimumab therapy could serve as inexpensive biomarkers of clinical outcome.
18	Swami et al., 2020 (30) Retrospective study	169 patients with unresectable, advanced, or metastatic cutaneous melanomas, received anti-PD-1 therapies.	Baseline eosinophil counts were not associated with PFS or OS in multivariable analysis.
19	Krückel et al., 2019 (5) Retrospective study	56 patients with metastatic melanoma	Patients with low eosinophil cationic protein at initial diagnosis of metastatic disease had a longer survival in comparison with patients with high eosinophil cationic protein.
20	Nakamura et al., 2019 (31) Retrospective study	45 patients with melanoma, received nivolumab or pembrolizumab	Baseline absolute eosinophil count was positively associated with occurrence of endocrine immune-related adverse events. This study did not find a significant association between eosinophils and OS, PFS or therapy response.
21	Lang et al., 2018 (32) Retrospective study	80 patients with unresectable advanced cutaneous stage IIIC or IV melanoma, received vemurafenib or ipilimumab	Patients who achieved long-term survival showed significant increase in eosinophils between beginning of therapy and last infusion of ipilimumab. There were no such findings in the vemurafenib group. For ipilimumab, an increase in lymphocytes and eosinophils during course of treatment correlated with long-term survival.
22	Gambichler et al., 2018 (33) Retrospective study	52 Patients with stage IIIc or IV melanoma, received ipilimumab	Baseline (pretreatment) eosinophils and eosinophil/lymphocyte ratio were not significantly associated with overall response rates, progression-free survival, melanoma-specific survival or AEs.
23	Rosner et al., 2018 (34) Retrospective study	209 patients with unresectable stage III or IV melanoma, received nivolumab plus ipilimumab	Higher relative lymphocytes, relative eosinophils, and relative basophils were significantly correlated with improved OS. However, there was no statistically significant correlation between absolute eosinophilic count and OS.
24	Moreira et al., 2017 (35) Retrospective study	173 patients with metastatic melanoma; 86 with immunotherapy (ipilimumab, pembrolizumab, nivolumab or combination therapy), 87 without immunotherapy	Eosinophilia is a prognostic marker in patients with metastatic melanoma.
25	Heppt et al., 2017 (3) Retrospective study	101 patients with metastatic uveal melanoma, received either PD-1 inhibitor monotherapy (n=86) or combined PD-1 inhibitor and ipilimumab (n=15)	Normal serum levels of LDH and CRP and a high relative eosinophil count may help identify patients with better prognosis.
26	Fujisawa et al., 2017 (36) Retrospective study	101 patients with unresectable or stage IV melanoma, treated with nivolumab	The increased absolute lymphocyte and eosinophil count did not correlate with the occurrence of severe irAEs.
27	de Coaña et al., 2017 (37) Prospective study	43 advanced melanoma patients, received ipilimumab	The absolute counts of eosinophils in peripheral blood at the 3- and 9-week time points were significantly higher in patients that presented immune related adverse events of any grade.
28	Ferrucci et al., 2017 (38) Retrospective study	244 patients with advanced melanoma, received chemotherapy (n = 116) or anti-CTLA-4 therapy (n = 128)	Relative eosinophil counts ≥ 1.5% was associated with a favorable outcome for patients receiving anti-CTLA-4, but not with the prognosis of patients receiving chemotherapy. Patients with relative eosinophil counts ≥ 1.5% tended to have a delayed disease progression compared to patients with REC < 1.5% if they were treated anti-CTLA-4, but not if they received chemotherapy, although differences were not significant in multivariate analysis.
29	Weide et al., 2016 (6) Retrospective study	616 patients with unresectable stage III or stage IV melanoma, received pembrolizumab	A significant positive correlation with OS for high absolute and relative eosinophil counts were observed, in the patients with melanoma treated with pembrolizumab.
30	Martens, et al., 2016 (39) Retrospective study	615 patients with advanced melanoma patients, received ipilimumab in 2 independent cohorts	Absolute and relative eosinophil counts were positively correlated with survival.
31	Khoja et al., 2016 (40) Retrospective study	183 patients with stage III-IV melanoma, received ipilimumab	No associations of eosinophil to lymphocyte ratio with toxicity or response were found.
32	Gebhardt et al., 2015 (41) Retrospective study	59 patients with stage IV melanoma, received ipilimumab	An early increase in eosinophil count during the treatment with ipilimumab was associated with an improved clinical response. In addition, the content of eotaxin-1 in serum from nonresponding melanoma patients was significantly lower than before the therapy. This chemokine is considered to play a critical role in the eosinophil recruitment.
33	Delyon et al., 2013 (42) Prospective study	73 Patients with unresectable stage III or IV melanoma with at least one previous line of chemotherapy, received ipilimumab.	Eosinophil counts at the first course were not associated with OS. Biological data such as lymphocyte and eosinophil counts at the time of the second ipilimumab infusion appear to be early markers associated with better OS. In addition, an increase >100/mm3 in the absolute eosinophil count was associated with longer survival as well as an increase >100% in the absolute eosinophil count between the first two ipilimumab courses.

# ICI, Immune Checkpoint Inhibitor.

*TCGA, The Cancer Genome Atlas.

### 3.1 Role of eosinophils independent of therapy type or treatment initiation

Four publications have investigated the prognostic value of eosinophils, independent of patient therapy. In a recent study by Zhang et al. (25) 80 uveal melanoma patients in The Cancer Genome Atlas (TCGA) were classified into two immune subgroups of the tumor microenvironment. Class 1 has low immune infiltration, contains memory B-cells, T helper-2 cells, T helper-17 cells, natural killer cells and eosinophilic granulocytes, and has a better prognosis. CD8^+^ T cells, T helper-1 cells, myeloid-derived suppressor cells, and dendritic cells are enriched in class 2, which has strong cytolytic activity, high expression of immune checkpoint genes and poor outcome (25). In another study, Wagner et al. (28) evaluated the impact of peripheral blood leukocytes on OS in 1412 patients with melanoma (stage I-II) who underwent sentinel lymph node biopsy. They concluded that peripheral blood leukocytes are independently associated with OS in patients with stage I-II melanoma and should be considered as a prognostic marker. An absolute eosinophil count ≤200/µL was associated with a decreased OS in their study (28). In addition, Krückel et al. (5) investigated eosinophil cationic protein (ECP) as an early prognostic marker in 56 patients with metastatic melanoma. This marker mediates anti-cancer effects, such as tissue remodeling and cytotoxic activity. Therefore, they concluded that ECP is a novel prognostic serum marker for the outcome of melanoma patients that is independent of LDH and easy to perform in clinical practice (5). Similarly, Moreira et al. (35) investigated whether eosinophilia is a prognostic marker in 173 patients with metastatic melanoma. They observed that melanoma patients with eosinophilia at any point in the course of their disease showed a trend toward longer survival, regardless of their therapy (35).

### 3.2 Eosinophilic granulocytes and anti-PD1 monotherapy (pembrolizumab, nivolumab)

In a recent study by Bai et al. (26), pre-treatment eosinophilic blood counts were negatively correlated with OS in 89 patients with advanced melanoma treated with the anti-PD-1 monotherapy from two prospective clinical trials. In contrast, Swame et al. and Nakamura et al. (30, 31) failed to demonstrate an association between OS and eosinophilic blood counts. Similarly, in the study by Kurzhals et al. (7), lymphocyte and eosinophil counts at baseline and during immunotherapy were not associated with disease recurrence. However, in three further studies, high relative eosinophil blood counts correlated with an improved OS (3, 6, 35). Similarly, Amman et al. (20) found a positive correlation between increased tumor-infiltrating eosinophils and T cells and delayed melanoma progression. Furthermore, high baseline eosinophil count, serum ECP, and eosinophil peroxidase levels were associated with prolonged progression-free survival (PFS) in metastatic melanoma under immune checkpoint inhibition (20). Pozorski et al. (15) also recently demonstrated that the baseline neutrophil/eosinophil ratio may be a novel prognostic marker for advanced melanoma patients receiving anti-PD-1-based therapies.

Simon et al. (27) suggested that eosinophil levels may be a novel predictive marker for melanoma patients who may benefit from the immunotherapy, since clinical responses to immune checkpoint inhibitor treatment were associated with peripheral blood eosinophil accumulation (27). Similarly, Kartolo et al. (23) and Ohashi et al. (18) were able to show that eosinophilia on immunotherapy could be a favorable sign for advanced malignant melanoma. However, the retrospective study of melanoma patients receiving either nivolumab or pembrolizumab by Nakamura et al. (31) did not find a correlation between relative and absolute eosinophil blood counts and treatment response. In addition, the study by Bai et al. (26) found that the low early-on-/pre-treatment fold change in eosinophil count was not significantly associated with a better response to treatment.

Another retrospective study showed that patients with a high absolute pre-treatment eosinophilic count (EC) had a higher risk of immune-related adverse events (irAEs) (31). In line with this, Tasaki et al (14), recently showed that elevated eosinophils prior to two courses of treatment may be a predictor of immune-related adverse events in various cancers treated with different immune checkpoint inhibitors. However, Fujisawa et al. (36) found no significant correlation between the relative counts of eosinophils (REC) and irAEs, in their retrospective analysis of 101 patients with unresectable or stage IV melanoma, treated with nivolumab.

### 3.3 Eosinophilic granulocytes and CTLA4 monotherapy (ipilimumab)

In the study by Balatoni et al. (29) baseline absolute eosinophil counts >0.1 G/L were significantly associated with worse PFS in 47 patients with advanced melanoma treated with ipilimumab. However, Marlens et al. (39) reported that absolute and relative eosinophil counts were positively correlated with survival following ipilimumab treatment in their patients with advanced melanoma. In a similar retrospective study by Ferrucci et al. (38), a relative eosinophil count ≥ 1.5% was associated with a favorable outcome in patients receiving anti-CTLA4. However, in other studies, the baseline eosinophil count was not associated with OS (33, 42).

Machiraju et al. (24) observed a significant increase in absolute blood eosinophils with anti-CTLA4 treatment. Further studies reported that this increase in eosinophils during treatment with anti-CTLA4 monotherapy was a predictor of better OS (32, 41, 42).

In contrast, in a study of 43 patients with advanced melanoma receiving ipilimumab, absolute peripheral blood eosinophil counts at 3 and 9 weeks were significantly higher in patients who presented with irAEs of any grade (37). Nevertheless, Khoja et al. (40) reported that the eosinophil/lymphocyte ratio was not associated with toxicity or therapeutic response.

### 3.4 Eosinophilic granulocytes and anti-PD1/Anti-CTLA4 combination therapy

Some studies have shown a significant correlation between REC and OS in patients treated with an anti-PD1/anti-CTLA4 combination therapy (3, 27, 34). These studies showed that higher REC was as a prognostic marker for better survival (3, 27, 34). In addition, Machiraju et al. (24) observed in their retrospective study that patients generally experience an increase in peripheral blood eosinophils during treatment. Interestingly, Simon et al. (27) showed that treatment responders had a significantly higher increase in peripheral blood eosinophils during treatment than non-responders.

### 3.5 Eosinophilic granulocytes and other melanoma therapies

Ferrucci et al. (38) showed that EC in patients underwent chemotherapy was not associated with PFS or OS in patients with advanced melanoma.

Lang et al. (32) retrospectively investigated the role of eosinophils during treatment with B-Raf proto-oncogene, serine/threonine kinase (BRAF) inhibitors. No significant association was found between the eosinophil count and OS. They also found no correlation between eosinophils and long-term survival (32). Wendlinger et al. (21) demonstrated that high pre-treatment eosinophil counts in patients with advanced melanoma were associated with a significantly improved response to MAPK signaling pathway inhibitors (MAPKi). Functionally, eosinophils have potent cytotoxicity against melanoma cells that can be enhanced by MAPKi (21).

A single phase II cohort investigated the prognostic role of eosinophils in 41 patients with advanced melanoma treated with a combination therapy of BEMPEG (a PEGylated interleukin-2 [IL-2] prodrug) and nivolumab (22). The findings showed that a strong increase in eosinophils during the first eight days of treatment was a positive prognostic marker for better response to therapy, but not for a better PFS (22). In another study, prolonged PFS and OS were significantly associated with an increase in absolute peripheral blood eosinophil count during additional intralesional treatment with IL-2 in patients with locoregional progression on immunotherapy (19).

## 4 Discussion

The potential importance of baseline biomarkers present in both the blood and the tumor microenvironment for guiding pretreatment selection and predicting prognosis in melanoma patients has received considerable attention in the literature (19). The important beneficial effect of eosinophils and recent clinical studies suggest that eosinophils may have a significant impact on tumor progression in several types of cancer (43–45). Studies have reported that, depending on the type of cancer, the presence of eosinophils may either be beneficial for survival, or worsen the outcome. For example, some studies suggest a beneficial effect of high eosinophil blood counts in colon cancer, prostate cancer, breast cancer and melanoma (35, 46–49). In contrast, patients with Hodgkin’s lymphoma who have an increase in eosinophils seem to have a worse survival rate (50). Eosinophils are capable of phagocytosis and can express MHC-II on their surface, and can migrate to regional lymph nodes (51–53). Depending on the stimulus, they can activate innate and adaptive immunity, communicate with T cells and mast cells, and participate in the initiation and maintenance of an inflammatory state (54). In addition to the known and described properties of eosinophilic granulocytes, their effect and role in malignancies has mostly been studied *in vitro* or in animal models. Eosinophils play a role in tissue remodeling and cell turnover in both homeostasis and disease (45). In the context of tumors, eosinophils are often found in areas of tissue necrosis, and there is evidence that eosinophils can exert a cytotoxic effect on tumor cells, both *in vitro* and *in vivo*. Finally, tumor-associated tissue eosinophilia (TATE) appears to be generally protective (45). Several studies have shown an improved prognosis with TATE or evidence of eosinophil degranulation in various types of solid tumors (35, 45–47). In a large national cohort of metastatic solid tumor could show better OS correlated with increased eosinophil count (16). Moreira et al. and Wagner et al. (28, 35) supported this statement specifically for melanoma by demonstrating defined baseline eosinophilic granulocytes as a positive predictive marker for improved OS, independent of therapy. Zhang et al. (25) also showed that the presence of eosinophilic granulocytes in TME was associated with a better prognosis of choroidal melanoma.

Several factors might generally influence the response to ICI treatment, including tumor mutational burden, tumor microenvironment, and stool microbiome (55). Serum markers such as lactate dehydrogenase, PD-L1 expression on melanoma cells, microsatellite instability, and the composition of circulating blood cells such as lymphocytes, neutrophils, and eosinophils, either individually or in combination, have potential predictive value (56). However, studies evaluating the prognostic value of peripheral eosinophilic blood count in patients treated with various systemic therapies for OS have shown heterogeneous results. In addition, there were significant differences in the design and analytic methods of the available studies. They included patients with various melanoma subtypes at different melanoma stages, making it difficult to compare their results. There were also significant differences among studies concerning the association between eosinophilic granulocyte count and treatment response. Despite the extremely heterogeneous data, eosinophils could serve as a prognostic marker in immunotherapy of melanoma, given their effects on the TME and the relationship between TME and ICI-therapy response (27, 57). In a retrospective cohort based on a pharmacovigilance registry that included 909 patients with various tumors receiving anti-PD-1 or anti-PD-L1 therapy, 2.8% of patients were found to have immune-related eosinophilia, the majority of whom were being treated for advanced melanoma (58). Analysis of inflammatory mediators showed in a recent study that IL-16 levels tend to be associated with the frequency of circulating eosinophils (27). Eosinophils are a source of IL-16, which is a chemoattractant for both lymphocytes and eosinophils (27, 59). Furthermore, eosinophils have been shown to attract CD8^+^ T cells to the tumor microenvironment in the absence of regulatory T cells, in a melanoma mouse model (27). In a similar study, eosinophils were shown to enhance CD8^+^ T cells activation and improve the response to immunotherapy in breast cancer (60). This is important because previous publications found that tumor infiltration by CD8^+^ T cells was enhanced in responders before and during ICI treatment (27, 61). In addition, eosinophils from ICI-treated patients were shown to be enriched for IFN-γ response signatures and IL-2 signaling (27). IFN-γ signaling was found to be essential for the beneficial effect of PD-1 inhibition (27, 61). Nevertheless, not all eosinophil effects are solely due to ICI therapy. Future studies should include melanoma patients undergoing different treatments to better understand the exact role of eosinophils (27).

In spite of possible association between the occurrence of immune-related adverse events and improved OS (17), irAEs can profoundly affect nearly every organ system, resulting in severe to fatal toxicities that require discontinuation of ICI therapy in a significant proportion of patients (15). Several factors have been implicated in the occurrence of irAEs in patients receiving ICIs, including younger age, higher BMI, gender, smoking history, the presence of multiple chronic diseases, pre-existing autoimmune conditions and chronic use of certain drugs (15, 62). Potential biomarkers for irAEs include circulating blood counts, cytokines, autoantibodies, HLA genotypes, microRNA, gene expression profiling, and serum proteins (62). Although pre-treatment eosinophil count was not associated with the occurrence of irAE, in the study by Mehra et al. (17), elevated eosinophils prior to treatment have recently been shown to be a predictor of immune-related adverse events in several cancers treated with different ICIs (14). Similarly, in a recent cohort of patients with metastatic renal cell carcinoma treated with ipilimumab and nivolumab, an elevated eosinophil level 2 weeks after treatment may be an effective biomarker for ≥grade 2 irAEs (63).

In conclusion, eosinophilic granulocytes and their secreted proteins can be used as prognostic biomarkers for patients with melanoma. The role of eosinophils in melanoma is being elucidated. Eosinophilic granulocytes appear to play an important role in melanoma and cancer and are a potentially valuable biomarker for predicting response to ICIs, but their exact role remains unclear. This is because current knowledge in this area is based on mostly retrospective studies with heterogeneous study designs (including patient populations, treatment regimens, follow-up protocols, statistical analysis methods and cut-off values), leading to inconsistent and sometimes even controversial results. In addition, the other possible cause of hypereosinophilia should be considered in more detail. We hope that the current review will encourage cancer specialists to investigate the impact of local and/or peripheral eosinophilia on survival in melanoma patients in a well-designed, standardized prospective study.

## Author contributions

CB: Conceptualization, Data curation, Writing – original draft, Writing – review & editing. RH: Conceptualization, Data curation, Methodology, Writing – original draft, Writing – review & editing. SS: Conceptualization, Data curation, Methodology, Supervision, Writing – original draft, Writing – review & editing.
